# Do Power Outages Impact Mental Health? Empirical evidence from Maryland

**DOI:** 10.21203/rs.3.rs-9163667/v1

**Published:** 2026-03-27

**Authors:** Meng Feng, Yueming (Lucy) Qiu, Jie Chen, Jiehong Lou, Yi David Wang

**Affiliations:** 1 School of Public Policy, University of Maryland College Park, College Park, MD, USA.; 2 School of Public Health, University of Maryland College Park, MD, USA..; 3 Department of Economics, Virginia Polytechnic Institute and State University, Blacksburg, VA

## Abstract

Power outages are becoming more frequent in the United States, yet their mental health consequences remain under-identified in clinical data. Maryland is a particularly relevant setting given its mental health service utilization rate of 42.2 per 1,000 population—nearly twice the national average—and its aging grid infrastructure under increasing climate stress. We combine high-frequency power outage records (15-minute intervals) with administrative inpatient claims from the Maryland Healthcare Cost and Utilization Project State Inpatient Database to estimate how outages affect mental-health-related hospitalizations from 2018 to 2023. In the baseline, we define severe outage exposure as the count of episodes exceeding the 75^th^ percentile of outage intensity and lasting at least one hour. Higher outage exposure is consistently associated with increased mental health hospitalization rates (IRR (Incidence Rate Ratio) ≈ 1.017, approximately a 1.7% increase). This association is robust to alternative severeity cutoffs (50^th^ and 90^th^ percentile), with effects concentrated in moderate-duration disruptions (1–3 hours) rather than prolonged outages. Instrumental-variable models using three grid infrastructure measures—transformer density, substation density, and transmission line density—indicate larger causal effects (IRR ≈ 1.35–1.52) than conventional fixed-effects models. Heterogeneity shows that effects are concentrated in large metropolitan areas and lower-poverty areas. Because hospitalization-based measures reflect both underlying need and access to care, these estimates likely understate the true population burden, particularly in underserved communities. These findings position grid reliability as a public health concern and highlight the importance of reducing recurrent short-duration outages, strengthening continuity of mental health care and medication access, and integrating health considerations into reliability planning for outage-prone communities.

## Introduction

Power outages are an increasingly pervasive and intensifying threat to public health in the United States (Chrobak, 2020; B. Li et al., 2026; McBrien et al., 2026), yet their impact on mental health remains insufficiently understood. In states such as Maryland, aging grid infrastructure poses a growing risk of power outages, while climate-driven extreme heat may further exacerbate this vulnerability by raising cooling demand, stressing grid performance, and worsening existing energy affordability burdens for low-income households (Maryland Energy Administration, 2024a; Maryland Office of People’s Counsel, 2022). Power outages disrupt mental healthcare delivery through multiple, interrelated pathways. Telehealth services may become unavailable, transportation to in-person appointments can be impaired, and pharmacy closures may hinder timely medication refills. During widespread outages, mental health crisis hotlines are often inaccessible when demand surges (Janzen, 2025; Sugg et al., 2023), while emergency psychiatric services face increased strain due to system-wide overload. Such mental health shocks may manifest as stress reactions, anxiety, exacerbation of psychotic disorders, or substance-use crises, yet most existing studies rely on survey methods to identify mental health issues without looking at actual clinical data.

Power outages could be life-threatening, particularly for individuals who depend on electricity for medical purposes (Molinari et al., 2017). Climate change is expected to increase the frequency, intensity, and duration of extreme weather events, which – compounded by aging infrastructure – may result in more severe and prolonged power disruptions (Panteli & Mancarella, 2015). As the frequency and duration of power outage continue to rise, medically vulnerable populations – particularly users of electricity-dependent durable medical equipment – are increasingly at risk, with disparities observed across socioeconomic and racial lines (Casey et al., 2021). In 2012, an estimated 685,000 individuals in the US relied on electricity-dependent medical devices while living at home (Molinari et al., 2017). Using a comparable Medicare-based measure from the U.S. Department of Health and Human Services (HHS) empower Program, beneficiaries with claims for electricity-dependent durable medical equipment identified about 3.0 million beneficiaries in December 2025 (U.S. Department of Health and Human Services, 2025), although the underlying population frame differs. From 2016 to 2025, Maryland’s HHS empower count for claims for electricity-dependent durable medical equipment increased from 28,939 to 35,575 when compared consistently in August of each year (+22.9%) (U.S. Department of Health and Human Services, 2025). These individuals face acute health risks during power disruptions and are not consistently integrated into emergency preparedness systems, increasing their vulnerability to both physical and psychological distress. Studies of Winter Storm Uri’s mental health impacts found high rates of psychological distress. 18% of 790 surveyed Texas residents reported post-traumatic stress; those experiencing both power and water outages combined with high COVID-19 impacts had 7.7 times higher odds of PTS (post-traumatic stress)(S. E. Grineski et al., 2022).

Maryland provides a particularly relevant setting for this study because its aging electricity infrastructure and increasing climate stress: Maryland’s electric grid has been described as “battered and getting worse” by state officials (Walton, 2024), with outages increasing due to aging infrastructure and more frequent severe weather events (Maryland Energy Administration, 2024b). In recent years, Maryland has retired 6,000 megawatts of power generation capacity while adding only 1,600 megawatts (MarylandReporter.com, 2026). PJM (Pennsylvania-New Jersey-Maryland) Interconnection has noted that “no major high-voltage lines constructed in recent memory” in Maryland as of 2024, leaving its transmission infrastructure increasingly strained (Kurtz, 2024). Consistent with this trend, the frequency of outage episodes lasting at least one hour in our data increased at an average annual rate of approximately 10% over the study period (2018–2023). The state encompasses both dense metropolitan corridors in the Baltimore–Washington region and rural and coastal communities on the Eastern Shore and in Western Maryland, creating substantial geographic heterogeneity in infrastructure exposure and healthcare access: approximately two-thirds of the ZIP codes in our sample (321 of 490) fall within large metropolitan areas (population ≥ 1 million), while the remaining third encompasses smaller metro areas and rural communities. In addition, this study uses high-resolution ZIP code-level outage data, whereas much of the prior literature relies on county-level datasets (Flores et al., 2023a; Oak Ridge National Laboratory (ORNL), 2025; PowerOutage.com, n.d.), allowing more precise estimation of localized exposure and community-level heterogeneity. When linked to Healthcare Cost and Utilization Project (HCUP) State Inpatient Databases (SID) administrative records, these data make Maryland an empirical setting for identifying the effects of power outages on clinically diagnosed mental health outcomes.

Prolonged power outages not only threaten public health in socioeconomically disadvantaged communities but also involve longer restoration delays during extreme weather events for vulnerable populations (Mitsova et al., 2018). In the U.S., the severe outages in California, Texas, and Louisiana in 2020 and 2021 caused by extreme weather were estimated to cost $2 billion, $80–130 billion, and $31–44 billion, respectively (Qiu et al., 2024). Recent evidence suggests that power outage burdens are unevenly distributed across communities. (Bhattacharyya & Hastak, 2023) found that ZIP codes with lower median household income experienced more frequent outages—a disparity the authors attributed to spatial differences in grid infrastructure vulnerability—while outage durations were shorter in areas with critical facilities such as hospitals and fire stations, consistent with utilities’ practice of prioritizing restoration in those areas. (Macmillan et al., 2023) found that areas with a high share of minority populations were more than four times more likely to suffer a blackout, and that low-income communities experience more frequent outages and less reliable electricity service. These disparities may be further compounded by aging electrical grid infrastructure; today over 70% of the US electrical grid is over 25 years old, increasing the system’s vulnerability to extreme weather events and other outage causes (U.S. Department of Energy, Grid Deployment Office, 2023).

The prevalence of mental health disorders in the United States has been surging over the last few years, affecting more than 22% of the adult population (Substance Abuse and Mental Health Services Administration (SAMHSA), 2023). Maryland faces a particularly acute mental health burden: the state’s mental health service utilization rate reached 42.2 per 1,000 population in 2023 – nearly twice the national average of 24.5 per 1,000 – with over 260,000 individuals receiving state mental health services, 78% of whome met the federal definition for serious mental illness or serious emotional disturben (Substance Abuse and Mental Health Services Administration (SAMHSA), 2024). Maryland’s Medicaid-funded share of mental health clients (97%) also substantially exceeds the national average (76%), reflecting both higher need and greater reliance on public insurance for behaviroal health care. In the US, major depressive disorder (MDD) affected 19.8 million adults in 2019, with an economic burden of $333.7 billion (Greenberg et al., 2023). Power outages are associated with psychological stress, anxiety, and disruption of daily life and communication (Andresen et al., 2023; Rubin & Rogers, 2019). Evidence from the 2021 Texas winter storm and power crisis also suggests that mental health effects can continue after the immediate event (Sugg et al., 2023). More broadly, recent evidence from Germany shows that energy poverty is strongly associated with deteriorations in mental rather than physical health, suggesting that inadequate access to reliable energy services can directly harm psychological well-being (Buchner & Rehm, 2025). As distress worsens, some patients could shift to emergency or inpatient care. Because mental and substance use-related hospital care is resource intensive, greater hospital utilization can increase healthcare system burden and spending (Owens et al., 2019). This pattern is consistent with broader evidence that serious mental illness carries substantial long-term lifetime economic costs (Seabury et al., 2019).

Despite growing evidence, important gaps remain. Many studies rely on self-reported, short-term distress rather than clinically diagnosed outcomes. (Hwong et al., 2022) conducted a scoping review of 56 empirical studies on climate change and mental health; 23 (41% of studies) relied on self-reported survey data which focused on self-reported distress shortly after immediate extreme weather events, which may not capture clinically diagnosed or longer-term mental health impacts. In addition, most existing work provide limited causal identification of the independent effect of power outages, because outages frequently co-occur with extreme weather events that affect mental health through separate pathways.

This study provides the first empirical evidence of power outages on mental health using high resolution power outage data and actual patient-level clinical data — the administrative inpatient claims in Maryland, and addresses these two gaps. First, we leverage patient-level administrative inpatient records from the HCUP State Inpatient Database (2018–2023), linkedin with high-resolution power outage data, to measure clinically diagnosed mental health hospitalizations rather than self-reported distress. Using multi-year administrative hospitalization data, we find that more frequent outage episodes of moderate duration (1–3 hours), rather than prolonged events, are associated with approximately a 1–2% increase in the rate of hospitalizations with a primary mental health diagnosis, measured at the ZIP code level. Second, to address endogeneity, we implement an instrumental variable strategy based on pre-existing electricity grid infrastructure - specifically, transformer density (distribution layer), substation density, and high-voltage transmission line density (transmission layer) – which exploit variation in the physical structure of the grid to identify a causal effect of power outages on mental health hospitalization rates. In addition, we integrate infrastructure stressors with social vulnerability factors and test their interaction effects on mental health risk, using ZIP code and time fixed effects, with results that are robust across specifications and stronger in more vulnerable communities.

Our findings contribute to the literature by shifting attention from rare, large-scale blackout events to more frequent, short-duration outages that characterize everyday grid operations. While often treated as routine operational issues, these interruptions are shown to be associated with measurable changes in mental health outcomes. At the same time, the heterogeneity patterns indicate that observed hospitalization effects are shaped by both underlying need and access to care. This implies that outage-related mental health burdens may be systematically under-recorded in communities with more limited healthcare access, highlighting a potential gap between observed outcomes and true population impacts.

### Our Contribution

Extreme weather events and power outages frequently co-occur across U.S. regions (Do et al., 2023), making it difficult to attribute mental health impacts to outages alone. Although both are linked to adverse mental health outcomes, they likely operate through distinct pathways: extreme weather, particularly high temperatures, can affect mental health through physiological channels such as thermoregulatory stress and sleep disruption (Basu et al., 2018; D. Li et al., 2023; Meadows et al., 2024), whereas power outages may affect mental health through infrastructure-dependent disruptions, including loss of thermal comfort, disrupted routines, and reduced access to social support (Casey et al., 2020; Rubin & Rogers, 2019), as well as interruptions in telehealth, medication access, and crisis support (S. Grineski et al., 2023; Sugg et al., 2023). However, prior empirical studies (Supplementary Information N1) have largely focused on disaster contexts in which weather extremes and outages occur simultaneously, limiting causal identification of the independent effect of outages (S. E. Grineski et al., 2022; Lin et al., 2016, 2021; Sheridan et al., 2021).

Our study makes the following contributions. Most of the prior literature (See Supplementary Information N1 for a detailed literature review) have largely examined the combined effects of co-occurring hazards rather than isolating the role of power outages, and have often relied on short-term event-based data that do not distinguish emergency department discharges from inpatient psychiatric admissions. This study addresses these limitations in two ways. First, unlike most prior studies that mainly relied on survey-based data to measure mental health (Hwong et al., 2022) rather than using clinical and administrative data, as we do here, our study leverages comprehensive administrative data from the Maryland HCUP SID (State Inpatient Database), allowing us to quantify the mental health burden of power outages more precisely by tracking post-ED hospitalization outcomes and to control for a richer set of temporal and geographic covariates. Administrative hospital data offer several advantages: they capture objectively diagnosed conditions using standardized International Classification of Diseases, 10^th^ Revision (ICD-10-CM, Appendix A) coding, provide population-level coverage of all inpatient admissions within the state. Self-reported mental health measures, such as those collected from surveys, are prone to reporting bias and may not accurately represent the severity of mental health disorders, as they are influenced by respondents’ perceptions and their willingness to disclose mental health issues. These surveys are inadequate for capturing the time impact of power outages on a large scale. To address these limitations, our analysis focuses on patients whose primary diagnosis is a mental health condition, including all diagnoses listed in (Appendix A). The primary diagnosis reflects the main reason for admission or treatment, which allows us to isolate acute mental health cases directly attributable to the underlying condition (cases where mental health is the main and underlying condition of the patient) rather than secondary or comorbid pathways. This approach thus provides a cleaner attribution of mental health outcomes to power outage events, minimizing confounding from unrelated physical health factors.

Second, estimating the causal impact of power outages on mental health poses significant challenges, primarily due to confounding factors—both observed and unobserved—that affect both exposure and outcomes. Using a large and statewide hospital admission database that covers the entire state of Maryland, this study (1) evaluates the causal impact of power outage on mental health hospitalizations as primary diagnosis; (2) investigates whether the relationship between outages and mental health varies across sociodemographic groups. To identify the causal effect of power outages on mental health admissions, we employ an identification strategy that accounts for multiple potential confounding pathways. Specifically, we adjust for time-varying weather conditions, demographic factors, include ZIP-code and time fixed effects, and implement an instrumental-variable (IV) approach based on electricity-grid infrastructure (e.g., transformer density and distribution-line density; with alternative specifications using substation density and high-voltage transmission-line density) to address potential endogeneity in outage exposure.

## Results

### Baseline estimates: power outages and mental health hospitalizations

**Figure 1.**
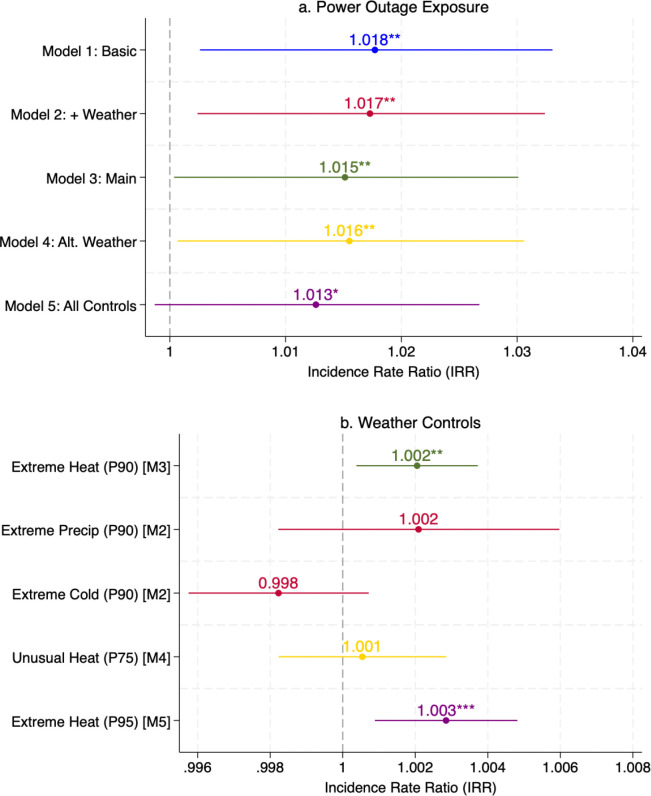

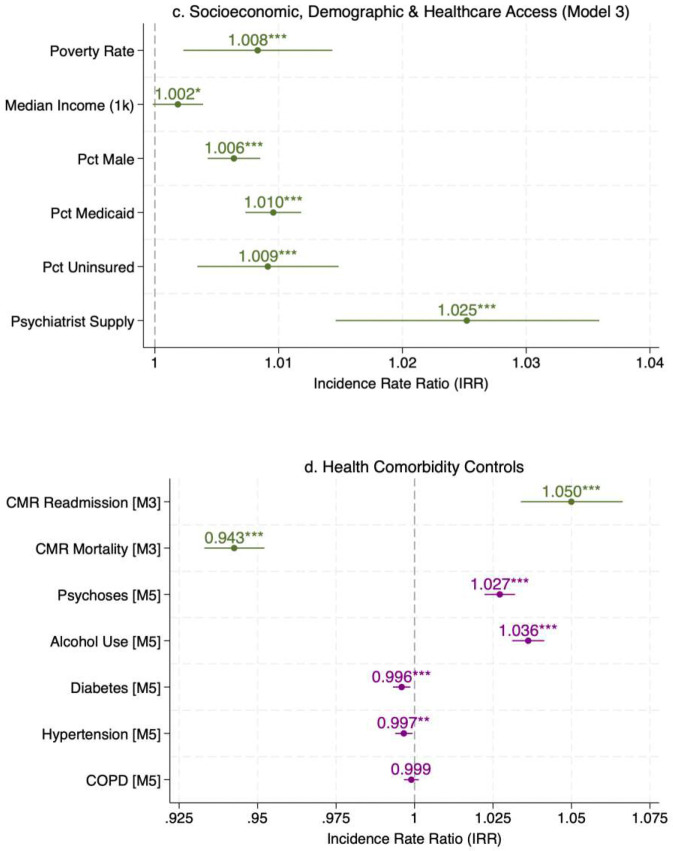
Baseline PPML coefficient estimates – mental health hospitalizations and power outages Note: Each panel presents incidence rate ratios (IRRs) with 95% confidence intervals from PPML-HDFE regressions. Panel (a) shows the main effect of power outage exposure (ln_outage_1hr_p75) across five model specifications. Panel (b) shows weather controls from respective model specifications. Panel (c) shows socioeconomic, demographic, and healthcare access controls from Model 3 (M3). Panel (d) shows health comorbidity controls (CMR (Clinical Risk Model) indices from Model 3; disease prevalence from Model 5). Model 1 (M1) includes poverty rate, median household income, and case-mix-adjusted comorbidity and mortality indices (CMR). Model (M2) adds extreme heat, extreme precipitation, and extreme cold days (all at the 90^th^ percentile threshold). Model 3 (M3) retains extreme heat (90^th^ percentile) and adds demographic controls (percent male, percent Black, percent Asian), insurance coverage (Medicaid share, uninsured share), and psychiatrist supply. Model 4 (M4) is identical to M3 but replaces the 90th percentile heat threshold with the 75th percentile. Model 5 (M5) uses the 95th percentile heat threshold, replaces the CMR comorbidity index with disease-specific prevalence indicators (psychoses, alcohol use disorders, diabetes, hypertension, COPD), and retains the CMR mortality index. All models include ZIP code and Year×Quarter fixed effects with total population as exposure. Standard errors clustered at ZIP code level. * p < 0.10, ** p < 0.05, *** p < 0.01. See Supplementary Table S14 for full regression results.

Figure 1 presents regression results from Poisson Pseudo-Maximum Likelihood models with high-dimensional fixed effects (PPML-HDFE), which regress ZIP-quarter-level mental health hospitalization rates on log-transformed power outage exposure – measured as the number of outage episodes per ZIP-quarter that lasted at least one hour and affected more than the 75^th^ percentile of customers (see Power Outage Data in the Methods section and Appendix B for variable definition and construction details) – across five progressively richer specifications that sequentially add weather, socioeconomic, demographic, and health-system covariates. All models include ZIP code and year-by-quarter fixed effects to account for time-invariant local characteristics and absorb shocks common to all ZIP codes in a given year-quarter. The identification strategy relies on within-ZIP variation over time in power out exposure, under the assumption that – conditional on fixed effects and observed covariates-the timing of outage is exogenous to local mental health needs, reporting behavior, or hospital utilization. A positive, stable association across all specifications is the main finding of the baseline analysis.

Figure 1 presents the PPML-HDFE regression results across five specifications, with all models including ZIP code and year-by-quarter fixed effects and using total population as the exposure term. The positive association between power outage exposure and mental health hospitalizations is robust across all specifications (Panel a). The estimated IRR for ln_outage_1hr_p75 (the natural log of one plus the count of outage episodes lasting at least one hour and exceeding the 75^th^ percentile of outage intensity; see Power Outage Data in the Methods section and Appendix B for variable definition and construction details) ranges from 1.013 to 1.017, indicating that a one-unit increase in log outage exposure—approximately a 2.7-fold increase in severe outage episodes (e.g., from 5 to 14 per quarter)—is associated with a 1.3–1.7% increase in mental-health-related hospitalization rates. Beginning with a basic specification including socioeconomic controls and comorbidity indices (Model 1, IRR = 1.017, p < 0.05), the effect remains stable as we add weather controls (Model 2, IRR = 1.017, p < 0.05), demographic and healthcare access variables (Model 3, IRR = 1.015, p < 0.05), an alternative heat threshold at the 75^th^ percentile (Model 4, IRR = 1.016, p<0.05), and a set of disease-specific comorbidity indicators (Model 5, IRR = 1.013, p < 0.10). This stability indicates that the estimated effect is robust to the inclusion of observable confounders.

Among the environmental controls, extreme heat emerges as an significant risk factor for mental health admissions (Panel b). Extreme heat days at the 90th percentile show a significant positive association with hospitalization rates (IRR = 1.003, p < 0.01 in Model 2; IRR = 1.002, p < 0.05 in Model 3), and the effect remains significant when using the more stringent 95th percentile threshold in Model 5 (IRR = 1.003, p < 0.01). In contrast, extreme precipitation and extreme cold days show no statistically significant associations in Model 2, and the lower heat threshold at the 75th percentile is also not significant in Model 4, suggesting that only more extreme temperatures meaningfully affect mental health outcomes. Importantly, the power outage effect remains significant even after controlling for these weather variables, confirming that outages exert an independent impact on mental health beyond weather-driven stress. Beyond the main outage effect, the control variables help characterize factors shaping mental health hospitalization patterns across Maryland ZIP codes (Figure 1, Panel c-d; see Supplementary Table S14 for full results). A clear pattern emerges around healthcare access. Areas with more psychiatric providers per capita are associated with higher – not lower – mental health admission rates (IRR = 1.025–1.028, p<0.01), consistent with greater case identification and referral rather than higher underlying burden.

Similarly, ZIP codes with higher Medicaid enrollment (IRR = 1.006–1.010, p < 0.01) and uninsured rates (IRR = 1.005–1.009, p < 0.01 in main models) show significantly more admissions. This pattern is consistent with the idea that populations with more limited access to outpatient or preventive care may rely more heavily on crisis-driven inpatient services. Socioeconomic disadvantage is consistently associated with higher hospitalization rates. For every one percentage point increase in the poverty rate, mental health admissions increase by 0.6–0.8% (IRR = 1.006–1.008), while median household income shows a small positive association in Models 3 and 4 (IRR = 1.002, p < 0.10)—a pattern that may reflect better access to care and diagnosis in higher-income areas rather than a higher underlying burden of mental illness. Demographic composition also matters: ZIP codes with a higher share of male residents show consistently higher admission rates (IRR = 1.005–1.006, p < 0.01). Areas with larger Asian populations have substantially lower admission rates (IRR = 0.331–0.340, p < 0.05), even after controlling for other factors. The percentage of Black residents is not statistically significant after controlling for other variables (IRR = 0.970–0.983).

The health comorbidity controls capture an important dimension of population vulnerability. Areas with higher average CMR (Clinical Risk Model) readmission risk—capturing a greater burden of underlying comorbidities (see Appendix E for details)—exhibit significantly higher mental health hospitalization rates (IRR = 1.049–1.050, p < 0.01 in Models 1–4). This pattern suggests that populations with more complex underlying health conditions may be more vulnerable to mental health–related hospitalizations. Conversely, areas with higher mortality risk exhibit lower mental health hospitalization rates (IRR = 0.939–0.972, p < 0.01). When disease-specific indicators are included in Model 5, areas with higher prevalence of psychoses (IRR = 1.027, p < 0.01) and alcohol-related disorders (IRR = 1.036, p < 0.01) show significantly higher admission rates, whereas physical conditions such as diabetes with complications (IRR = 0.996, p < 0.01) and hypertension with complications (IRR = 0.997, p < 0.05) are negatively associated. COPD (Chronic Obstructive Pulmonary Disease) shows no significant association (IRR = 0.999).

### Alternative definitions of outage exposure: robustness and sensitivity

**Figure 2:**
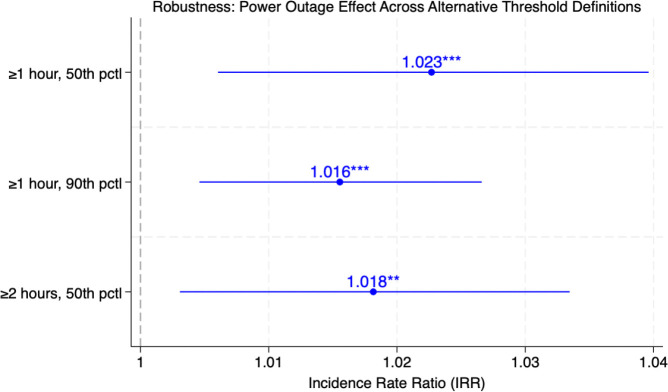
Robustness – power outage effect across alternative threshold definitions Note: Each point shows the incidence rate ratio (IRR) from a separate PPML regression using alternative outage threshold definitions. Horizontal bars represent 95% confidence intervals. ** p < 0.05, *** p < 0.01. All models include ZIP code and Year x Quarter fixed effects with the full set of controls. See Supplementary Table S15 for full results.

We assess whether the estimated outage–mental health relationship is sensitive to alternative definitions of outage exposure. In the baseline specification, outage exposure is defined as episodes lasting at least one hour and exceeding the 75th percentile of severity. To evaluate sensitivity, Figure 2 checks the robustness under three alternative threshold definitions that vary both the minimum duration and severity cutoff. All specifications retain the full set of controls, ZIP code and Year × Quarter fixed effects, and population exposure.

The results (Figure 2) are consistent across specifications. Defining outages as events lasting at least one hour above the 50th percentile of severity yields an IRR of 1.023 (p < 0.01), implying a 2.3% increase in mental health hospitalization rates per unit increase in log outage exposure. Increasing the severity threshold to the 90th percentile produces a slightly smaller but more precisely estimated effect (IRR = 1.016, p < 0.01). Extending the minimum duration to two hours while maintaining the 50th percentile cutoff yields a nearly identical estimate (IRR = 1.018, p < 0.05). Across all definitions, point estimates fall within a narrow 1.6–2.3% range, indicating that the estimated relationship is not driven by any single threshold choice. Full regression results are reported in Supplementary Table S15.

**Figure 3:**
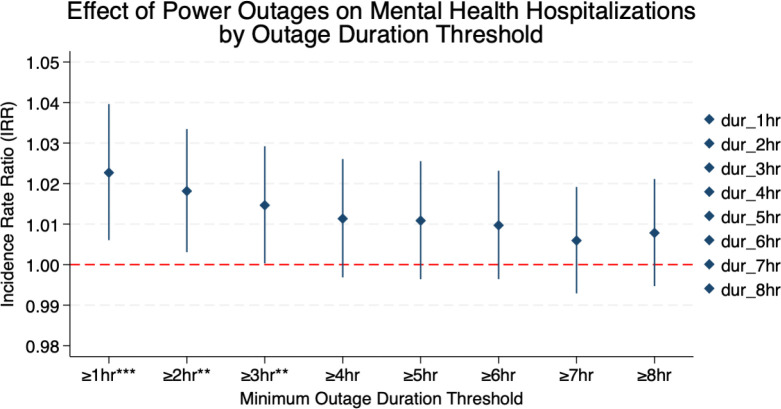
Sensitivity Test Note: Each point shows the incidence rate ratio (IRR) from a separate PPML regression. The outage measure is defined as ln(1 + episodes), where episodes are outage events lasting ≥ X hours and above the 50th percentile of severity within each ZIP–quarter. 95% confidence intervals are shown. ** p < 0.05, * p < 0.1. All models include ZIP and Year × Quarter fixed effects. Summary of the statistics for outage episodes by duration threshold (Log-transformed) can be found in the supplementary table S16.

We further examine sensitivity to the minimum outage duration threshold by estimating separate PPML regressions with outage exposure defined as ln(1 + episodes), where episodes correspond to outages lasting at least X hours and exceeding the 50th percentile of severity within each ZIP–quarter (Figure 3) (see Power Outage Data in the Methods section and Appendix B for details on exposure variable construction). The estimated effects are largest for shorter-duration outages. Outages lasting at least one hour yield an IRR of approximately 1.023 (p < 0.01). The estimated effect declines gradually as the duration threshold increases, with IRRs of about 1.018 (p < 0.05) at two hours and 1.015 (p < 0.05) at three hours. For thresholds of four hours and above, the estimates are smaller in magnitude and no longer statistically distinguishable from zero, with confidence intervals overlapping the null value of 1. Shorter outages are substantially more frequent than longer-duration events (Supplementary Table S16), suggesting that the loss of statistical significance at higher thresholds likely reflects reduced statistical significance at higher thresholds likely reflects reduced statistical power rather than a reversal of the underlying relationship.

Taken together, these robustness and sensitivity analyses indicate that the positive association between power outages and mental health hospitalizations is not driven by any single threshold definition. The results are consistent across alternative severity cutoffs and duration thresholds, with effects concentrated among relatively frequent, moderate-duration outages (1–3 hours), rather than rare, prolonged events (Figure 3). This pattern suggests that even short-duration outages can accumulate over time and contribute meaningfully to mental health burdens in affected communities. Overall, the findings highlight the importance of outage frequency, in addition to event severity, in understanding the mental health impacts of power infrastructure disruptions.

As an additional specification check, replacing ZIP-code fixed effects with city-by-year fixed effects – which absorb city-level time-varying unobservables such as local policy and economic shifts - yields a consistent and somewhat larger estimate (IRR = 1.031, 95% CI: 1.007–1.056, p = 0.012), confirming that the positive association is not driven by city-level confounders that evolve over the study period (see Supplementary Table S17 for full result).

### Effect modification by extreme heat

To examine whether the association between power outages and mental health hospitalizations is amplified during periods of extreme heat, we estimate an augmented PPML-HDFE model that incorporates an interaction between log outage exposure and extreme heat days, both mean-centered at the sample mean. This specification tests whether concurrent thermal stress and electricity disruption compound the psychological burden beyond the sum of their individual effects.

The interaction term (Table 1) is not statistically significant (IRR = 1.000, 95% CI: 1.000–1.001, p = 0.434; joint test: χ^2^(1) = 0.61, p = 0.434), suggesting that the mental health impact of power outages does not significantly differ across levels of extreme heat exposure. The main effect of outage exposure remains positive (IRR = 1.015, p = 0.050), consistent with the baseline specification, and extreme heat days are independently associated with higher mental health hospitalization rates (IRR = 1.002, p = 0.025).

### Instrumental Variable Results

To address potential endogeneity in outage exposure, we implement an instrumental variable (IV) approach using three measures of electricity grid infrastructure – transformer density, substation density, and high-voltage transmission line density (see Appendix F for details on construction of the instrumental variables) – as instruments for power outage exposure. Our IV estimates confirm a positive and statistically significant effect of power outage exposure on mental health hospitalizations, consistent in direction with the baseline PPML results, lending support to a causal interpretation that power outages adversely affect mental health outcomes (see Methods for detailed instrument construction, identification assumptions, and the physical architecture of the power grid).

The first-stage regressions confirm (Table 2) that grid infrastructure predicts outage exposure. Both transmission-level and distribution-level infrastructure densities are significantly associated with the frequency of severe outage episodes. This supports the relevance condition: areas with grid structures that are more susceptible to cascading or equipment-level failures indeed experience more outage exposure.

Table 2 presents IV Poisson estimates using three alternative instrument sets. In Column (1), transformer density alone serves as the excluded instrument and is negatively associated with outage intensity (−0.116, p < 0.01), consistent with denser local networks being better able to localize faults; the F-statistic of 9.80 indicates adequate instrument strength. Column (2) uses upstream transmission infrastructure—substation density and transmission line density—both positively associated with outage exposure (1.706 and 0.189, both p < 0.01), reflecting the greater cascade failure risk in highly interconnected bulk power networks; the F-statistic of 21.32 confirms strong instruments. Column (3) combines all three variables: transformer density remains negatively associated (−0.102, p < 0.01), while substation density and transmission line density remain positive (1.640 and 0.177, both p < 0.01), with a joint F-statistic of 19.76.

In the second stage, all three specifications yield positive and statistically significant effects of outage exposure on mental health hospitalizations, with consistent direction across instrument sets. The coefficient on ln_outage_1hr_p75 is 0.420 (p < 0.01) using the transformer IV (Column 1), 0.301 (p < 0.10) using transmission IVs (Column 2), and 0.380 (p < 0.01) using all three IVs (Column 3). Because the model is estimated in exponential mean form, these coefficients imply that a one-unit increase in log outage exposure is associated with approximately a 35–52% increase in mental-health-related hospitalizations (IRR ≈ 1.35–1.52). The Hansen J test for overidentification does not reject instrument validity in either of the overidentified specifications (p = 0.383 for Column 2; p = 0.556 for Column 3), strongly supporting the exogeneity of the instruments. The consistency of the estimates across downstream and upstream infrastructure measures—which capture distinct outage mechanisms—provides robust evidence that power outages increase mental health hospitalizations.

While the IV estimates are substantially larger than the PPML estimates, the implied effect size remains plausible when translated into absolute terms. The IV coefficient of 0.380 (IRR = 1.46) implies that a one-unit increase in log outage exposure—equivalent to approximately tripling the number of outage episodes (e.g., from 3 to 8 episodes per quarter)—is associated with a 46% increase in mental health hospitalizations. Given the mean baseline rate of 16.4 hospitalizations per ZIP-quarter, this translates to approximately 8 additional cases (16.4 × 0.46 ≈ 7.5). However, the distribution of hospitalizations is highly right-skewed, with a median of only 4 cases per ZIP-quarter. For a typical ZIP code at the median, a 46% increase would imply approximately 1.8 additional hospitalizations. Moreover, the IV estimates (IRR ranging from 1.35 to 1.52 across instrument sets) are substantially larger than the baseline PPML estimates (IRR ≈ 1.013 – 1.017). A cross-sectional Poisson specification without ZIP fixed effects but with the full set of controls yields an IRR of only 1.037, suggesting that the absence of ZIP fixed effects contributes minimally to the magnitude difference. We therefore interpret the PPML estimates as a conservative lower bound and the IV estimates as an upper bound on the true effect; both approaches consistently confirm a positive and statistically significant effect of power outage exposure on mental health hospitalizations. The IV results are robust to alternative outage definitions. Across seven specifications varying episode duration (1–8 hours) and severity thresholds (P50 and P75), all IV estimates remain positive, with five reaching statistical significance (IRR = 1.46–2.01), particularly at shorter duration thresholds. All Hansen J tests pass (p > 0.40; see Supplementary Information N2 and Supplementary Figure N1 for details).

### Healthcare Access, Socioeconomic Status, Racial Composition, and Seasonal Heterogeneity

To assess whether the relationship between power outages and mental health hospitalizations varies across communities, we examine heterogeneity along five dimensions: urban–rural classification, poverty level, Medicaid coverage, seasonality, and gender composition. Urban–rural status is classified using the USDA Rural-Urban Continuum Codes (RUCC): large metropolitan areas (RUCC=1, counties in metropolitan areas with a population of 1 million or more) versus all other areas. High-poverty ZIP codes are defined using the U.S. Census Bureau threshold of a poverty rate ≥20%. High Medicaid coverage is defined as ZIP codes where at least one quarter of hospitalized patients are covered by Medicaid, a threshold close to the national average of approximately 25% (Hulver et al., 2025). Warm season includes April–September (quarters 2–3) and cold season includes October–March (quarters 1 and 4). Gender composition is defined using a median split of the ZIP code–level share of male patients.

**Figure 4:**
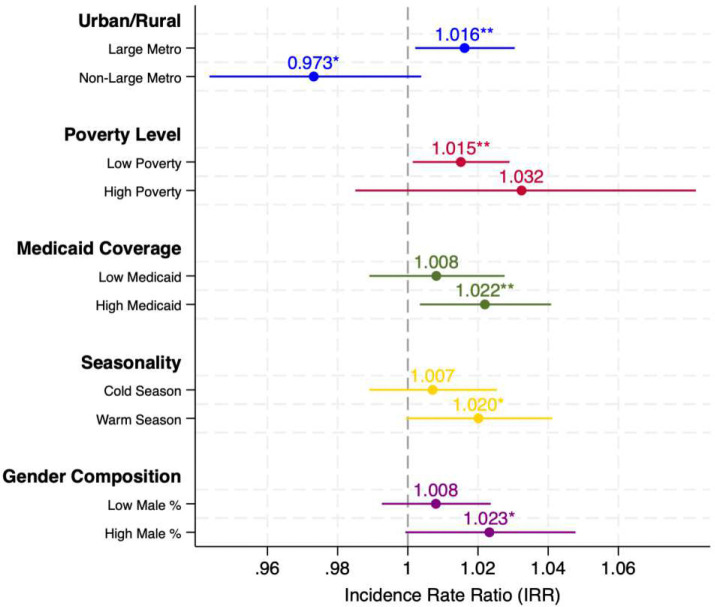
Heterogeneity Analysis: Power Outage Effects on Mental Health Hospitalizations Note: Each point represents the incidence rate ratio (IRR) for the outage variable (ln_outage_1hr_p75) from separate PPML-HDFE regressions estimated within subgroups, with 95% confidence intervals shown as horizontal bars. The dashed vertical line marks IRR = 1 (no effect). Urban–rural status is classified using USDA Rural-Urban Continuum Codes: Large Metro areas (RUCC = 1, counties in metropolitan areas with ⩾1 million population) versus all other areas. High Poverty is defined using the U.S. Census Bureau threshold of a poverty rate ⩾20%. Medicaid coverage subgroups are defined by a median split of the ZIP code – level share of Medicaid-covered discharges, with High Medicaid defined as ⩾25%. Warm Season includes Q2 – Q3 (April – September) and Cold Season includes Q1 and Q4 (January – March and October – December) (see Supplementary Tables S18). All models include ZIP code and Year × Quarter fixed effects, and use total population as the exposure variable. Controls include extreme heat days, substance use prevalence, comorbidity burden, unemployment, racial composition, insurance coverage, and psychiatrist supply, with minor adjustments by dimension (e.g., extreme heat days are excluded from seasonal models to avoid collinearity;Medicaid and uninsured shares are replaced by median income in Medicaid coverage models to avoid mechanical correlation with the grouping variable). Standard errors are clustered at the ZIP code level. * p < 0.10, ** p < 0.05.

Based on urban–rural status, the estimated effects are concentrated in large metropolitan areas. In large metro area ZIP codes, outages are associated with a 1.6% increase in mental health hospitalizations (IRR = 1.016, p < 0.05) (Figure 4). In contrast, the estimate for non-large-metro areas is negative and marginally significant (IRR = 0.973, p < 0.10). Formal tests confirm that this is the only dimension along which the subgroup difference is statistically significant (z-test p = 0.013; interaction p = 0.015) (see Supplementary Table S18). One plausible explanation is that residents in large metropolitan areas have better access to hospital-based care, including emergency and psychiatric services, making them more likely to seek treatment following outage-related stress. By contrast, residents in smaller metro and rural areas may face greater barriers to inpatient care or rely more on informal support networks, which could lead to fewer recorded hospitalizations even if underlying distress increases.

A similar pattern emerges when stratifying by poverty level using the U.S. Census Bureau threshold of 20%. The effect of outages is statistically significant in lower-poverty areas (IRR = 1.015, p < 0.05), while the point estimate in higher-poverty areas is larger but imprecise and not statistically significant (IRR = 1.032, p > 0.10), likely reflecting the relatively small number of ZIP codes exceeding the 20% poverty threshold in Maryland. The interaction model suggests a marginally significant difference between subgroups (p = 0.084), though the z-test does not confirm this (p = 0.499).

When stratifying by Medicaid coverage, ZIP codes with higher Medicaid coverage (≥25% of patients covered by Medicaid) show a significant association between outages and hospitalizations (IRR = 1.022, p < 0.05), whereas low-Medicaid areas show a smaller and non-significant effect (IRR = 1.008, p > 0.10). This pattern is consistent with greater vulnerability among communities with higher shares of publicly insured patients. However, formal tests indicate that this subgroup difference is not statistically significant (z-test p = 0.313; interaction p = 0.483). We also examine whether the outage effect varies by season. The estimate is larger in the warm season (IRR = 1.020, p < 0.10) than in the cold season (IRR = 1.007, p > 0.10), consistent with a heat-related mechanism in which outages during warmer months disrupt access to air conditioning, increasing thermal stress that may exacerbate mental health conditions. However, the seasonal difference is not statistically significant (z-test p = 0.353; interaction p = 0.223). Finally, when stratifying by gender composition (median split of the ZIP code–level share of male patients), the effect is somewhat larger in areas with a higher male share (IRR = 1.023, p < 0.10) than in areas with a lower male share (IRR = 1.008, p > 0.10), though the difference is not statistically significant (z-test p = 0.297; interaction p = 0.907).

## Conclusion and discussions

This study provides evidence of a positive causal relationship between power outages and mental health hospitalizations, using patient-level administrative data from Maryland (2018–2023) and multiple identification strategies. These findings carry implications beyond Maryland. Globally, mental disorders affected an estimated 970 million individuals in 2019 and remained among the top ten leading causes of disease burden (GBD 2019 Mental Disorders Collaborators, 2022). The full scale of this burden is likely underestimated: when premature mortality attributable to mental disorders is included alongside morbidity, an estimated 418 million disability-adjusted life years (DALYs) — approximately 16% of all global DALYs — can be attributed to mental disorders, with an associated economic cost of approximately USD 5 trillion (Arias et al., 2022). In the United States, more than 22% of the adult population is affected by mental health disorders (Substance Abuse and Mental Health Services Administration (SAMHSA), 2023), and economic losses attributable to mental disorders in high-income North America could account for up to 8% of gross domestic product (Arias et al., 2022).

Maryland presents a particularly acute case: its mental health service utilization rate (42.2 per 1,000 population) is nearly twice the national average (24.5 per 1,000) (Substance Abuse and Mental Health Services Administration (SAMHSA), 2024), indicating an already-elevated baseline demand for psychiatric care that additional outage-related hospitalizations may further strain. As aging infrastructure and increasingly frequent climate-related extremes place energy systems worldwide at growing risk of major outages (International Energy Agency, 2024; Tonne, 2026), the mental health consequences documented here are likely to extend to other regions facing similar grid vulnerabilities. Baseline PPML estimates indicate a modest increase in hospitalization rates associated with outage exposure, while IV estimates confirm a positive and statistically significant effect. Across specifications, the results are consistent in direction. Sensitivity analyses further show that the effect is driven primarily by short-duration outages (1–3 hours)—frequent disruptions that characterize everyday grid operations—rather than rare, prolonged blackout events.

These findings contribute to a growing literature linking power outages to adverse health outcomes. Prior work has documented mortality effects and mental health impacts during major disasters. However, existing literature largely focuses on extreme events and often cannot isolate the independent effect of outages from concurrent shocks. By contrast, our results provide causal evidence that even routine, everyday outages are associated with increases in hospitalizations for primary mental health conditions.

A key mechanism underlying this relationship is that short, recurrent outages are typically not treated as emergency events and therefore do not prompt broader response or support, leaving affected individuals to cope with repeated disruptions without additional assistance. Unlike large-scale blackouts, which often trigger broader responses such as shelters and crisis services, brief outages typically leave individuals—particularly those with pre-existing mental health conditions—without additional support. These disruptions can accumulate over time and worsen mental health, in some cases leading to hospitalization. While our data cannot identify the exact mechanisms, these effects are consistent with disruptions to care or medication, loss of heating or cooling, and accumulated psychological stress. We also find suggestive evidence that effects are somewhat stronger in the warm season, which may reflect additional stress from heat exposure, although this result is not robust and should be interpreted cautiously.

The heterogeneity results help clarify where these effects are most likely to be observed. Larger effects in urban ZIP codes, lower-poverty areas, and communities with lower Medicaid coverage suggest that outage-related distress is more likely to be captured in inpatient records where psychiatric care and referral capacity are available. This implies that hospitalization-based measures reflect not only underlying need but also access to care. As a result, our estimates likely understate the true population burden, particularly in underserved communities where impacts may go untreated or unrecorded.

These findings have important implications for infrastructure planning and regulatory decision-making. When outages generate additional demand for mental health care, the associated costs—borne by households, healthcare systems, and public payers—are not directly reflected in standard reliability metrics or routinely incorporated into infrastructure planning decisions. As a result, current approaches to evaluating grid reliability may undervalue the broader public health consequences of outages.

In this context, our results highlight the importance of considering frequent, short-duration interruptions—rather than focusing exclusively on rare, large-scale blackout events—when assessing reliability performance and planning investments. This pattern echoes (Bakke, 2016) contrast between headline large blackouts and the routine interruptions of everyday grid operation. As she notes, “outages of five minutes or less are actually more costly to the efficient running of our national economy, in part because they are so much more prevalent.” This perspective aligns with our finding that frequent short-duration disruptions, although often treated as minor events, can accumulate into meaningful health-related burdens.

From a policy perspective, the key implication is prioritization: reliability planning should place greater emphasis on reducing recurrent short outages that undermine day-to-day service continuity, especially in communities with higher vulnerability and lower coping capacity. These priorities are reinforced by the Maryland context: the state’s mental health service utilization rate is nearly twice the national average, and 97% of public mental health clients are Medicaid-funded, consistent with our finding that ZIP codes with higher Medicaid enrollment exhibit significantly higher hospitalization rates(Substance Abuse and Mental Health Services Administration (SAMHSA), 2024). Reducing outage frequency in these communities may help avoid additional strain on an already overburdened public behavioral health system. Taken together, these findings suggest that the social burden of grid unreliability is not limited to major blackout events; frequent routine interruptions may also carry meaningful health consequences and should be explicitly considered in planning and preparedness.

Several limitations should be noted. First, our outcome measure captures only inpatient admissions with a primary mental health diagnosis, and therefore reflects only a subset of individuals who access formal psychiatric care. Second, the analysis is limited to Maryland and may not generalize to regions with different infrastructure, climate conditions, or healthcare systems. Third, the IV estimates identify local average treatment effects and do not allow us to distinguish among specific behavioral or physiological pathways linking outages to mental health outcomes.

Looking forward, the relevance of these findings is likely to increase. Climate change is expected to raise the frequency and severity of extreme weather events that disrupt electricity systems, while the electrification of transportation and heating, together with growing electricity demand from data centers, is placing additional strain on grid infrastructure. Together, these trends imply that outage risk may rise at the same time that reliance on electricity becomes more critical for daily life and health. As a result, the health consequences of grid unreliability are unlikely to remain constant and may become more pronounced over time. For individuals managing chronic psychiatric conditions, even brief interruptions can disrupt medication refrigeration, telehealth access,and the environmental stability on which daily functioning depends. More broadly, these results suggest that grid reliability should be understood not only as an engineering or economic issue, but also as a public health concern. In particular, ensuring continuity of mental health services and medication access during outages should be integrated into emergency preparedness and grid investment planning. Recognizing health-related impacts in reliability planning and investment evaluation may lead to different assessments of the benefits of reliability improvements, particularly for measures that reduce frequent, short-duration interruptions.

## Methods

### Data

This study combines data from multiple sources to analyze the relationship between power outages and mental health-related hospital and emergency visits in Maryland. The primary dataset includes administrative hospital discharge data from the Healthcare Cost and Utilization Project (HCUP), specifically the State Inpatient Database (SID) for Maryland, covering the years 2018–2023. We focus on visit-level (per discharge level) records rather than patient-level data. HCUP does not allow tracking individual patients over time, which means longitudinal analysis at the individual level is not feasible. Furthermore, HCUP SID database captures only hospital-based encounters—inpatient hospitalizations—but does not include office-based care, such as visits to outpatient clinics, psychologists, or primary care physicians. As a result, this study likely underrepresents mild-to-moderate mental health cases that do not lead to hospital visits.

### Defining Mental Health Outcomes

Our primary outcome is inpatient admissions with a primary mental health diagnosis, identified in HCUP SID database using the principal diagnosis only (DX1), so that mental health is the main reason for hospitalization rather than a comorbidity. We classify diagnoses using ICD-10 mental health codes (refer to Appendix A for the details) and aggregate discharge records to the ZIP code-quarter level for Maryland (2018–2023). For each ZIP code-quarter, we construct “mh_primary_cases”, defined as the count of admissions with a primary mental health diagnosis.

The HCUP SID Maryland file (2018–2023) includes de-identified patient discharges with ZIPCODE identifiers and provides details such as age, gender, race/ethnicity, primary and secondary diagnoses, and the patient’s county. The HCUP SID does not include exact discharge dates; temporal information is limited to discharge month (DMONTH) and quarter (DQTR). For Maryland, only DQTR is available (Agency for Healthcare Research and Quality, 2020). Consequently, hospital discharge records are aggregated to the ZIP code–quarter level.

The sample includes 3,279,869 inpatient cases in Maryland (2018–2023). Of these, 209,988 (6.40%) had a mental health primary diagnosis, while 1,175,748 (35.85%) had mental health conditions only as secondary diagnoses, indicating that mental health is more often recorded as a comorbidity than the main reason for admission. Across any diagnosis position, substance use disorders were most common (19.60%), followed by depression (18.83%), anxiety (17.05%), psychotic disorders (2.94%), and stress-related disorders (2.63%); categories are not mutually exclusive. The main analysis uses primary-diagnosis mental health admissions only, which improves specificity but likely underestimates true ZIP code-level burden because inpatient data exclude undiagnosed and outpatient-only cases (Frank & Glied, 2023).

### Power Outage (PO) data

We reviewed prior studies to summarize how power outages have been defined in the literature using U.S.-based outage data. Most studies construct a binary exposure variable based on percentile thresholds of the proportion of customers affected by outages in a given geographic area. For example, (Lin et al., 2021; Sheridan et al., 2021; Xiao et al., 2021; Zhang et al., 2020) all classify a division-day as a PO day if PO coverage exceeds the 50^th^ percentile of its distribution, while (Deng et al., 2022) apply the 75^th^ percentile. In these studies, each day meeting the threshold is classified into mutually exclusive day types (e.g., PO only, hazard only, PO+hazard, neither) for use in daily time-series models. However, because our analysis is conducted at the quarterly level, a binary indicator would obscure meaningful variation: nearly all ZIP-quarters in our sample experience at least one outage, so a binary measure cannot distinguish a ZIP-quarter with one episode from another with dozens. We therefore use episode counts as a continuous exposure measure to capture the intensive margin of repeated disruption.

Our episode identification procedure draws on Do et al. (2023), who used data from PowerOutage.us at 10-minute, county-level resolution and defined outage events as continuous periods in which the proportion of customers without power met or exceeded 0.1% (the 90^th^ percentile of county-hourly outage rates). They classified events by duration, distinguishing 1+hour outages (which disrupt commerce and daily routines) and 8+ hour outages (a “medically relevant” duration exceeding the battery life of most electricity-dependent medical equipment and long enough for food, medication, and thermal regulation to be compromised).

PO records at the utility provider level were obtained from the Maryland State Department, recorded every 15 minutes from 2018 to 2023. Each record includes the timestamp of the PO, the provider, and the number of customers affected within each ZIP code in Maryland. Because many utilities do not report the total number of customers served at the ZIP level, we adopt the approach used by poweroutage.us (PowerOutage.com, n.d.) to approximate customer coverage. In this approach, “Customers Tracked” for each ZIP–provider pair is defined as the maximum number of customers affected observed historically (2014–2023), which is treated as a proxy for the total customer base in that area (see Appendix B for details). PO coverage is then calculated as the ratio of customers affected to the estimated total number of customers tracked:

$${OutageRate\;}_{i}=\frac{{\text{CustomersAffected}}_{\;i}}{{\text{CustomersTracked}}_{\text{zip}\;,\text{provider}}}$$

where Customers Tracked represents the maximum observed outage for each ZIP code-provider combination across the entire study period. A PO occurrence (Yes/No) is then defined as any 15-minute interval in which PO coverage exceeded the 75th percentile of the distribution among all intervals with nonzero outages (0.51% of customers affected at any given time point; such as the 75^th^ percentile). This threshold focuses on severe, large-scale events and reduce noise from minor disruptions, consistent with prior power outage definitions in the literature summarized in Table 3). Applying this criterion yields 2,003,452 observations (16.54% of total records) classified as PO occurrences and 151,276 distinct outage episodes identified across 1,191 ZIP codes.

To identify distinct outage events, consecutive flagged observations are grouped into continuous episodes, where observations separated by 25 minutes or less were considered part of the same episode (see Appendix B for the episode identification details). Our primary exposure variable in the baseline specification, “new_1hr_episodes_p75,” is defined as the number of outage episodes in ZIP code i and quarter t that last at least 1 hour and exceed the 75th percentile of outage intensity within the sample. The threshold (p75) indicates that only the most severe 25% of outage events—those with an outage rate above the 75th percentile—are included. Episodes are assigned to the quarter in which they begin, so that outage events spanning multiple quarters are counted only once rather than being double-counted. We construct the log-transformed measure (to normalize the right-skewed distribution and allows coefficients to be interpreted as approximate percentage changes) (Appendix B for detailed calculation of the power outage variable):

$$ln\;outage_{it}=ln\left(new\_1hr\_episodes\_p\;75_{it}+1\right)$$

where new_1hr_episodes_p75 it denotes the number of one-hour outage episodes at the 75th percentile threshold for ZIP code i in quarter t. The percentile-based severity threshold follows the power outage-health literature (Deng et al., 2022; Lin et al., 2021; Sheridan et al., 2021; Xiao et al., 2021; Zhang et al., 2020), while the episode identification procesure-flagging continuour periods where outage severeity exceeds a percentile cutoff and persists beyond a minium duration-parallels the methodology in Do et al 2023. The log transformation compresses the right-skewed distribution while The constant 1 is added to accommodate zero values. Results are robust to using untransformed episode counts in place of the log-transformed measure (IRR = 1.002, p < 0.10; Full results in the Supplementary Table S19), suggesting that the positive association does not depend on the functional form of the exposure variable. The magnitude is consistent with the log-transformed specification: a ZIP code moving from the 25th to the 75th percentile of outage exposure—approximately 6 additional episodes per quarter—would see a 1.2% increase in hospitalization risk (1.002^6^ ≈ 1.012), comparable to the baseline estimate from the log specification (IRR ≈ 1.015–1.017). A detailed description of the variable construction procedure is provided in Appendix B.

Due to data limitations, admission dates in the HCUP Maryland SID database are only available at the quarterly level, while power outage data are available at 15-minute intervals. We therefore aggregate both datasets to the ZIP code-quarter level for analysis in Maryland. The final dataset contains quarterly counts of outage episodes for each ZIP code, along with additional measures including episode counts at alternative duration thresholds (2–8 hours) for sensitivity and robustness checks.

### Control Variables and Integration with External Data

To improve causal inference and control for confounding factors, we integrate several external datasets with the HCUP Maryland SID.

**Socioeconomic characteristics** – including total population, poverty rate, unemployment rate, and median household income – were obtained from the five-year estimates of the U.S. Census Bureau’s American Community Survey (ACS) at the ZIP Code Tabulation Area (ZCTA) level. These variables capture community-level economic vulnerability that may influence both infrastructure reliability and healthcare utilization patterns.

**Healthcare access variables** were extracted from the Area Health Resources Files (AHRF), including psychiatrist supply per capita, hospital bed capacity, and primary care physician availability (see Appendix D for detailed variable definitions). These controls account for geographic variation in healthcare resources that may affect mental health service utilization independent of power outage exposure.

**Patient-level clinical characteristics** were aggregated from the HCUP Maryland SID to the ZIP-quarter level to control for underlying population health status. We include the Elixhauser Comorbidity Index to adjust for physical health conditions that may mediate outage effects (e.g., diabetes patients requiring insulin refrigeration, cardiac patients relying on medical devices), as well as baseline mental health burden measured by the prevalence of psychoses, alcohol-related disorders, and drug abuse (see Appendix E for comorbidity index construction).

**Weather variables** were incorporated to account for environmental factors that may jointly influence both power outages and mental health outcomes. We include measures of extreme heat days, extreme cold days, precipitation, and wind conditions as controls. These variables isolate the effect of power outages from the direct physiological and psychological impacts of adverse weather (see Appendix C for weather variable construction and threshold definitions).

The integration of these datasets allows us to control for environmental, socioeconomic, healthcare access, and clinical factors that may confound the relationship between power outages and mental health outcomes.

### Empirical strategy

We estimate the effect of power outage exposure on mental health admissions using Poisson Pseudo-Maximum Likelihood (PPML). This approach is well-suited for non-negative count outcomes, robust to overdispersion, and does not require strict distributional assumptions. The model includes total ZIP-quarter population as the exposure term, estimating per-capita admission rates.

We adopt the Poisson Pseudo-Maximum Likelihood (PPML) estimator rather than a log-linear OLS specification (e.g., reghdfe). This approach is well-established in the health economics literature for modeling hospitalization counts using administrative data. (Pickens et al., 2018, 2019) employ PPML to model hospital inpatient utilization rates and service demand following health care reform using the HCUP SID/SEDD database, with population as the exposure term—the same specification we adopt. More recently, (Graff Zivin et al., 2023) use a Poisson count data model—numerically equivalent to PPML—to estimate the effect of air pollution on influenza hospitalizations using HCUP administrative records at the county-month level. Our empirical setting closely mirrors these studies: all three use HCUP administrative hospitalization records aggregated to geographic-by-time units, with non-negative count outcomes and population-based exposure terms. Following the methodological rationale established in these studies, PPML is preferred over log-linear OLS for several reasons. First, as (Graff Zivin et al., 2023) note, “the PPML estimator performs well with a large number of zeros and over-or-underdispersion in the data”—both features present in our data, where many ZIP-quarters record zero mental health hospitalizations. A log-linear OLS approach would require transforming the outcome (e.g., log(y + 1)), which discards information from zeros and introduces bias. Second, the PPML estimator “yields consistent parameter estimates regardless of whether the dependent variable follows a Poisson distribution, so long as the conditional mean is properly specified,” and “the consistency properties of the estimator are retained” even when the variance assumption is violated (Pickens et al., 2018). This is particularly relevant in our setting, where hospitalization counts vary substantially across ZIP codes of different population sizes. By incorporating total ZIP-quarter population as an offset, our PPML specification directly estimates per-capita admission rates, making the coefficients interpretable as incidence rate ratios.

The baseline specification adopts a two-way fixed effects regression. ZIP code fixed effects account for time-invariant local characteristics such as geography, infrastructure, and demographic composition. Year-quarter fixed effects absorb shocks common to all areas, including seasonal trends and statewide policy or economic shifts. In other words, we assume that within a given ZIP code, any change in the frequency or intensity of outages over time is uncorrelated with unobserved determinants of mental health admissions in that area, after accounting for controls.

We include several observed time-varying covariates to further mitigate concerns about confounding. These include: socioeconomic and demographic factors (poverty rate, median household income, percent male, percent Black, percent Asian); insurance coverage (Medicaid share, uninsured share); healthcare supply (psychiatrists per 100,000 residents); and health vulnerability measures (case-mix-adjusted comorbidity and mortality indices). By including these covariates, we address the possibility that outage exposure may be correlated with underlying trends in local health vulnerability, access to care, or socioeconomic stressors that also influence mental health outcomes. The outcome is measured at the ZIP code-by-quarter level. Because we include total ZIP-quarter population as an offset (exposure) term, the PPML model estimates the admission rate per capita rather than raw counts. This makes the interpretation of coefficients comparable to rate-based outcomes often used in health services research (Correia et al., 2020; Santos Silva & Tenreyro, 2011, 2011).

Formally, the model is specified as:

$$\begin{array}{l} E\left[{\text{mh\_cases}\;}_{iqy}\right]\\ \;=exp\left(log\left({\;\text{population}\;}_{iqy}\right)+\beta_{1}log\left({\;\text{outage}\;}_{iqy}\right)+\beta_{2}{\;\text{heat}\;}_{iqy}\right.\\ \left.+X_{iqy}^{\prime }\gamma +\delta_{qy}+\theta_{i}\right) \end{array}$$

where:

- $mh\_cases_{\mathrm{iqy}}$ denotes the number of primary mental health hospitalizations in ZIP i, quarter q, year y;
- ${\mathrm{population}}_{iqy}$ is the total population used as an exposure term;
- ln (${outage}_{\text{iqy}}$)is the log-transformed count of significant outage episodes, using ln (episodes + 1)to address the zero-inflated and right-skewed nature of the data;
- ${heat}_{\mathrm{iqy}}$ is the number of extreme heat days (days above the ZIP-specific 90th percentile);
- $X_{iqy}$ is the vector of time-varying control variables;
- $\delta_{qy}$ are year-by-quarter fixed effects;
- $\theta_{i}$ are ZIP code fixed effects.

Standard errors are clustered at the ZIP code level to account for serial dependence within areas over time. We report incidence rate ratios (IRRs) rather than log coefficients to improve interpretability: an IRR greater than 1 indicates an increase in admission rates associated with the explanatory variable, while an IRR less than 1 indicates a reduction. For example, an IRR of 1.20 on implies that a one-unit increase in logged outage episodes is associated with a 20% increase in the rate of mental health admissions, holding other variables constant.

### Intrumental Variable Strategy

Although the baseline PPML regressions include ZIP code fixed effects, year-quarter fixed effects, and time-varying controls, unobserved factors correlated with both outage exposure and mental health hospitalization could still bias estimates. To address endogeneity concerns more directly, we implement an instrumental variable (IV) approach using electricity infrastructure density – substation density, transmission line density and transformer density – as instruments for power outage exposure (see Appendix F for details on construction of the instrumental variables).

**Figure 5:**
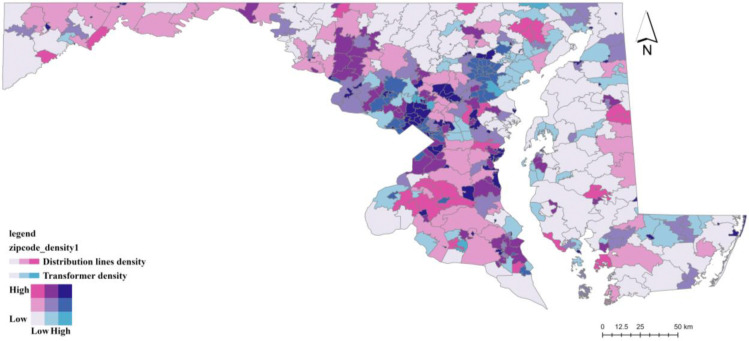
Spatial Distribution of Power Infrastructure Density Across Maryland Zip Codes Note: This figure presents a bivariate classification of distribution line density (pink) and transformer density (blue) at the ZIP code level. Combined colors (purple tones) indicate areas where both densities are high. Color intensity reflects relative density levels based on quantile classification, highlighting substantial geographic variation in local electricity infrastructure capacity across Maryland.

**Figure 6:**
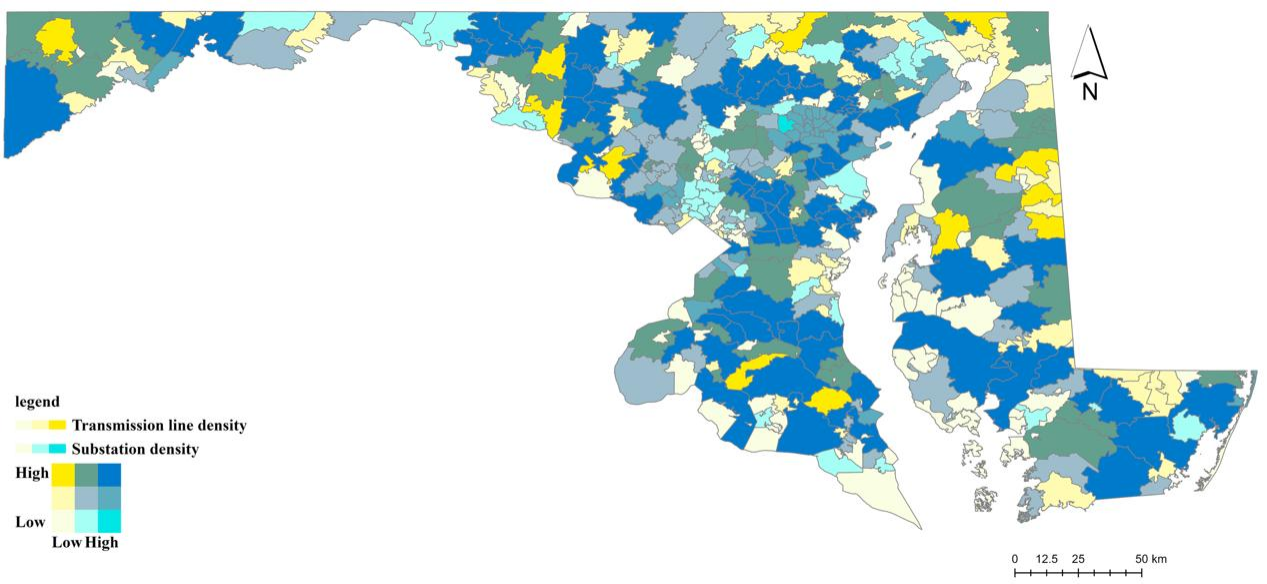
Spatial Distribution of Transmission Line and Substation Density Across Maryland ZIP Codes Note: Bivariate choropleth map displaying the joint distribution of transmission line density (yellow) and substation density (blue) across Maryland zip codes. Dark blue-green areas indicate high density of both infrastructure types; yellow areas have high transmission line but low substation density; blue areas have high substation but low transmission line density; pale/cream areas indicate low density of both.

The electric power grid operates as a hierarchical system with distinct transmission and distribution layers (see Supplementary Information N3 and Supplementary Figure N2). This architecture creates two vulnerability pathways that we exploit for identification. Failures in the upstream transmission network (substations and high-voltage lines) tend to produce widespread, cascading outages affecting large geographic areas, whereas failures in the downstream distribution network (transformers and distribution lines) typically generate more frequent but geographically localized disruptions. To capture these mechanisms, we construct instruments reflecting vulnerabilities at different grid layers. Transformer density measures the structure of the local distribution network serving end users, while substation density and high-voltage transmission line density capture exposure to disturbances in the regional bulk power system. The spatial distribution of these infrastructures varies substantially across Maryland ZIP codes (Figures 5 and 6) and is largely determined by long-term engineering, geographic, and regulatory considerations rather than short-run local health conditions (see Supplementary Information N3).

A limitation of the IV approach is that electrical grid infrastructure is inherently slow-changing capital stock. The instruments are therefore time-invariant and cannot be combined with ZIP code fixed effects. Our IV specification instead relies on cross-sectional identifying variation, controlling for time trends through year-quarter fixed effects and for ZIP-level confounders through a rich set of covariates.

## Supplementary Material

Supplementary Files

This is a list of supplementary files associated with this preprint. Click to download.

• AppendixandSupplementary.docx

## Figures and Tables

**Table 1:** Effect modification by extreme heat: Outage × Heat interaction (PPML-HDFE, ZIP-quarter level)

Variable	IRR	95% CI	p-value
Log outage hours (centered)	1.015	1.000–1.030	0.050
Extreme heat days (centered)	1.002	1.000–1.005	0.025
Outage × Heat (interaction)	1.000	1.000–1.001	0.434
N (observations)	8,152	—	—
Clusters (ZIP codes)	430	—	—

Notes: Estimates from PPML-HDFE model with ZIP code and Year × Quarter fixed effects and population offset. Log outage hours and extreme heat days are both mean-centered. IRR = incidence rate ratio. Joint test of interaction term: χ^2^(1) = 0.61, p = 0.4338. Full covariate set matches baseline model. Standard errors clustered at ZIP-code level.

**Table 2:** IV Poisson Estimates — Summary of Main Results

	(1) Transformer IVs	(2) Substation and transmission lines as IVs	(3) Transformer, Substation and transmission lines as IVs

**Panel A: Second Stage**			
ln_outage_1hr_p75	0.420*** (0.142)	0.301* (0.166)	0.380*** (0.120)

**Panel B: First Stage**			
dens_transformer	−0.116*** (0.037)	—	−0.102*** (0.029)
dens_substation	—	1.706*** (0.601)	1.640*** (0.595)
dens_transmission_line	—	0.189*** (0.067)	0.177*** (0.066)

Observations	8,204	8,204	8,204
First-stage F	9.80	21.32	19.76
First-stage p-value	< 0.001	< 0.001	< 0.001
Controls	Yes	Yes	Yes
Year FE	Yes	Yes	Yes
Hansen J p-value	-	0.383	0.556

Note: The table reports second-stage IV Poisson GMM estimates and first-stage coefficients for excluded instruments. Column (1) uses transformer density as the sole excluded instrument. Column (2) uses substation density and transmission line density as IVs. Column (3) uses all three infrastructure variables—transformer density, substation density, and transmission line density—as IVs. All models include year fixed effects and controls for extreme heat days, socioeconomic characteristics, insurance coverage, psychiatrist supply, comorbidity indices, and racial composition. Standard errors clustered at the ZIP code level.

* p<0.10

** p<0.05

*** p<0.01.

It should be noted that Column (1), using transformer density as the sole instrument, yields a first-stage F-statistic of 9.80—marginally below the conventional weak-instrument threshold of approximately 10 (Stock & Yogo, 2005) - indicating borderline instrument strength.

**Table 3:** Summary of Power Outage Definitions

References	Power Outage Definition	Location	Time Period	Resolution
(Zhang et al., 2020)	% customers affected, and Binary variable: yes/no %customers affected > 50^th^ percentile	New York State	2001–2013	Daily
(Lin et al., 2021)	Binary variable: yes/no %customers affected >50^th^ percentile	New York State	2001–2013	Daily
(Sheridan et al., 2021)	Binary variable: yes/no % customers affected > 50^th^ percentile	New York State	2005–2013	Daily
(Xiao et al., 2021)	Binary variable: yes/no % customers affected >50th percentile	New York State	Oct 29, 2012 to Nov 27, 2012	Daily
(Deng et al., 2022)	Binary variable: yes/no % customers affected >75^th^ percentile	New York State	2002–2018	Daily
(Do et al., 2023)	% customers affected	Nation wide, county level	2018–2020	Hourly
(Flores et al., 2023b)	% customers affected	New York State	2017–2020	Hourly

## Data Availability

The Healthcare Cost and Utilization Project State Inpatient Databases are restricted by a data use agreement, and cannot be made available without the express permission of the Agency for Healthcare Research and Quality.

## Code availability

All data are processed and analyzed in Stata (18MP) and Python. The figures are produced in Stata and ArcGIS Pro. The custom code is available on GitHub at https://github.com/ififif3333/PowerOutage_MentalHealth.
